# Editorial: Advances in Plastid Biology and Its Applications

**DOI:** 10.3389/fpls.2016.01396

**Published:** 2016-09-15

**Authors:** Niaz Ahmad, Steven J. Burgess, Brent L. Nielsen

**Affiliations:** ^1^Agricultural Biotechnology Division, National Institute for Biotechnology and Genetic EngineeringFaisalabad, Pakistan; ^2^Department of Plant Sciences, University of CambridgeCambridge, UK; ^3^Department of Microbiology and Molecular Biology, Brigham Young UniversityProvo, UT, USA

**Keywords:** plastids, plastid transformation, retrograde signaling, biopharming, metabolic engineering

Plastids originated from endosymbiosis around 1.5 billion years ago. They have been extensively studied to understand photosynthesis and other metabolic functions and to express foreign proteins, and knowledge about plastids has greatly increased. However, there are many aspects of plastid biology that remain unclear, and there have been difficulties in fully developing plastid transformation as an effective vehicle to express proteins. This research topic was launched to advance the knowledge of plastid biology, review recent progress, and address some of the challenges.

Tight coordination between plastid and nuclear genomes is essential for development and homeostasis in plant tissues. Bobik and Burch-Smith provide a detailed overview of this process including retrograde signaling between plastids and other organelles, plastid signaling in response to biotic and abiotic stress and the effect on the cell wall and intercellular symplasmic transport. By viewing chloroplast signaling in the context of the whole plant, they highlight the impact of chloroplast engineering on intracellular communication to avoid unintended consequences on growth and development.

An example of this concern is the alteration of carotenoid content of plants for the production of high value products. There is growing evidence that carotenoid cleavage products (apocarotenoids) can play an important role in modulating stress responses and impact upon plastid biogenesis; progress in identifying the signals and genes responsible is reviewed by Tian.

Chloroplast development from pro-plastids in angiosperms is dependent on light signaling pathways. Hills et al. demonstrated that classic plastid signaling also exists in gymnosperms but found that pine chloroplast biogenesis is light-independent. They investigated how light dependence might have evolved, and propose that suppression of photosynthetic gene responses to plastid signals in the dark occurred through recruitment of repressors of photomorphogenesis.

Organisms have evolved different mechanisms to cope with environmental stresses. The accumulation of osmoprotectants helps stabilize the active conformation of proteins and keeps cellular structures including membranes intact. D-arabitol accumulation in yeast provides protection against drought and salt stress. Khan et al. transferred the D-arabitol-mediated pathway into plants to test whether they could be made tolerant to drought and salinity. Overexpression of yeast arabitol dehydrogenase (ArDH)—an enzyme that reduces D-ribulose to D-arabitol—in tobacco chloroplasts conferred tolerance to NaCl up to 400 mM and 6% polyethylene glycol (PEG). This finding could have implications for developing stress-resilient crops to enhance yield.

Shimojima et al. utilized the inorganic phosphate (Pi) starvation response to stimulate triacylglycerol (TAG) accumulation in *Arabidopsis thaliana*. TAGs are useful as feedstocks for biofuel production or high value fatty acids, but are generally produced in seed tissues, which represent only a small proportion of overall plant biomass. TAG accumulation in vegetative tissues was increased in a starch deficient phosphoglucomutase mutant (*pgm-1*) background by overexpressing TAG synthesis enzymes under the low Pi inducible promoter of *monogalactosyldiacylglycerol synthase 3* (*MGD3*).

Infectious diseases continue to be a serious problem facing the growing human population. Significant research is focused on developing large-scale strategies for cost-effective production of therapeutics and vaccine antigens. Chloroplasts have the potential to express proteins at extraordinary levels, allowing plants to be used as green factories. Waheed et al. review the state-of the art of producing vaccines in plants via chloroplast transformation. They also discuss why chloroplast-made therapeutics have not reached the market despite a number of successful laboratory studies, and the major issues which should be addressed to fully exploit this technology.

Microalgal cells offer an alternative plastid expression system. Doron et al. provide a detailed overview of algal transformation systems, including techniques for transformation and genetic components, which encompasses selection markers, regulatory elements, promoters, inducible systems and plastid targeting signals.

Photosynthesis in C_3_ plants is relatively inefficient due to the partial loss of CO_2_ during recycling of 2-phosphoglycerate to 3-phosphoglycerate via photorespiration. Cyanobacteria and algae have CO_2_/bicarbonate transporters that concentrate CO_2_ around Rubisco, the CO_2_ concentrating mechanism (CCM). Expression of cyanobacterial CCM proteins in chloroplasts may improve photosynthesis in C_3_ plants. However, CCM proteins function in the inner envelope membrane (IEM) and localizing foreign proteins there has been difficult. Uehara et al. expressed two cyanobacterial bicarbonate transporters, BicA and SbtA, each tethered with an IEM transit peptide, in Arabidopsis chloroplasts. The effects on photosynthesis have not yet been examined, but the successful localization of the protein in the IEM is an important step toward improving photosynthesis.

Most early studies on plastid division examined mesophyll cells. Fujiwara et al. studied the effects of a mutation in the Arabidopsis AtMinE1 chloroplast division site determinant gene using CFP, YFP, and GFP fusions. The mutant had dramatic effects on plastid size and shape in epidermal cells but no significant differences in mesophyll cells. Mutants have enlarged plastids, and stromules and bulges emerging from the large plastids led to smaller subcompartments after FtsZ-mediated constriction. The larger plastids were unable to divide due to the inability to form a productive interaction with FtsZ. The results suggest that control of plastid division may differ among plant tissues.

Delfosse et al. reviewed the use of fluorescent proteins (FPs) to study chloroplast structure, extensions and stromules. The use of FPs has led to insights on interactions between chloroplasts, mitochondria and peroxisomes that suggest cooperation between organelles during photorespiration or in response to stress conditions or reactive oxygen species (ROS). Applications of FPs reviewed in this paper and a prior one (Hanson and Sattarzadeh, 2013) will lead to insights on other plastid types and interactions between intracellular organelles.

Plastid DNA levels change during plant development, with high amounts of DNA in young chloroplasts and significantly less in mature leaf cells. This should be considered in efforts to improve chloroplast transformation and plastid efficiency. Plants have two nuclear-encoded dual-localized DNA polymerases, and analysis of Arabidopsis mutants indicates that neither is totally essential for replication of either chloroplast or mitochondrial DNA. However, mutations in either result in decreased DNA levels in both organelles and slower growth depending on the mutant and age of the plant tissue tested (Morley and Nielsen).

In conclusion, this research topic summarizes current progress, challenges, and prospects for applications in plastid biology. Plastids vary in many significant characteristics depending on the tissue, age, and cell type. This includes changes in plastid division and structure, retrograde signaling, responses to stress, and DNA copy number. The complex signaling networks linking plastids to the nucleus and their evolution is only just starting to be understood. These reports underscore the importance of not over-interpreting findings on plastid properties or functions in specific plant tissues, and should be considered for full development of chloroplast genetic engineering for agricultural improvements and production of therapeutics.

## Author contributions

All authors listed, have made substantial, direct and intellectual contribution to the work, and approved it for publication.

## Funding

NA would like to thank HEC, Pakistan, for funding work in his laboratory; SB was supported by the European Union 3 to 4 project, and research in the BN lab was supported in part by a Brigham Young University Mentoring Environment Grant and by the National Institutes of Health (USA) (R15GM066787).

### Conflict of interest statement

The authors declare that the research was conducted in the absence of any commercial or financial relationships that could be construed as a potential conflict of interest.
